# *Mycobacterium tuberculosis* Infection in Free-Roaming Wild Asian Elephant

**DOI:** 10.3201/eid2303.161439

**Published:** 2017-03

**Authors:** Basavegowdanadoddi Marinaik Chandranaik, Beechagondahalli Papanna Shivashankar, Kunigal Srinivasa Umashankar, Poojappa Nandini, Papanna Giridhar, Somenahalli Munivenkatappa Byregowda, Basavegowdanadoddi Marinaik Shrinivasa

**Affiliations:** Institute of Animal Health and Veterinary Biologicals, Hebbal, Bangalore, India (B.M. Chandranaik, B.P.Shivashankar, P.Nandini, P.Giridhar, S.M.Byregowda);; Rajiv Gandhi National Park, Mysore District, Karnataka, India (K.S.Umashankar);; National Institute for Research in Tuberculosis, Chennai, India (B.M.Shrinivasa)

**Keywords:** Mycobacterium, tuberculosis and other mycobacteria, bacteria, Asian elephant, Elephas maximus, zoonoses, India

## Abstract

Postmortem examination of a wild Asian elephant at Rajiv Gandhi National Park, India, revealed nodular lesions, granulomas with central caseation, and acid-fast bacilli in the lungs. PCR and nucleotide sequencing confirmed the presence of *Mycobacterium tuberculosis*. This study indicates that wild elephants can harbor *M. tuberculosis* that can become fatal.

Tuberculosis (TB), a pandemic, highly contagious disease caused by *Mycobacterium tuberculosis* complex, has affected up to one third of the world’s human population. The South-East Asia Region (SEAR), which contains nearly one fourth of the world population, alone accounts for 38% of illnesses and 39% deaths caused by TB worldwide. India accounts for 58% of all forms of TB in SEAR and 55.6% of deaths caused by TB (excluding those among HIV-positive persons) in SEAR (*1*).

*M. bovis* is widespread in domestic animals and has been extensively documented in both captive and free-ranging wildlife. Although *M. tuberculosis* is primarily a pathogen of humans (*2*), it has been reported in zoo species (*3*,*4*) as well as in a formerly captive elephant in Africa (*5*) and a free-roaming elephant in Sri Lanka (*6*). We report the pathology and molecular characterization of *M. tuberculosis* in a wild Asian elephant (*Elephas maximus*) that had no known history of human contact and present implications for wildlife health.

In February 2016, a carcass of an ≈65-year-old free-roaming wild Asian elephant was found in the forest of Rajiv Gandhi National Park (RGNP), Karnataka, India. On postmortem examination, the lungs showed widely disseminated white-yellowish firm nodules with central caseous necrosis, distributed throughout the parenchyma (Figure, panel A). The bronchial and mediastinal lymph nodes were enlarged with nodular areas of caseous necrosis and calcification. Impression smears from the cut surfaces of lungs on staining by Ziehl-Neelsen method showed bundles of pink-stained acid-fast organisms.

**Figure:**
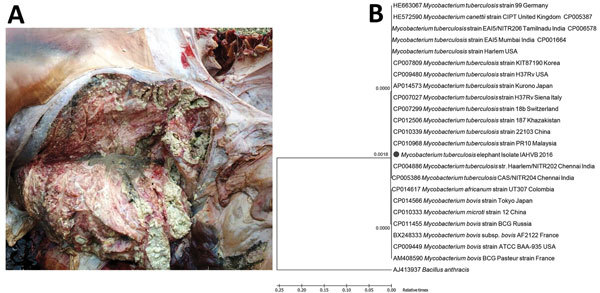
Findings from a deceased wild free-roaming Asian elephant (*Elephas maximus*) infected with *Mycobacterium tuberculosis*, Rajiv Gandhi National Park, Karnataka, India, 2016. A) Results of postmortem examination of the lungs. Note the widely disseminated white-yellowish firm nodules with central caseous necrosis. B) Phylogenetic analysis.

DNA extracted from the lung tissue were subjected to PCR targeting amplification of a conserved region on *M.* *tuberculosis* complex by using forward primer 5′-GACCACGACCGAAGAATCCGCTG-3′ and reverse primer 5′-CGGACAGGCCGAGTTTGGTCATC-3′ (*7*), which yielded a specific amplicon of 445 bp, indicating presence of a pathogenic mycobacterium. To detect *M. bovis*, we used forward primer 5′-CACCCCGATGATCTTCTGTT-3′ and reverse primer 5′-GCCAGTTTGCATTGCTATT-3′to amplify an 823-bp region on a 12.7-kb fragment of *M. bovis*. To detect *M. tuberculosis*, we used forward primer 5′-CACCCCGATGATCTTCTGTT-3′ and reverse primer 5′-GACCCGCTGATCAAAGGTAT-3′ to amplify a 389-bp region on a 12.7-kb fragment of *M. tuberculosis* (*7*). The PCR used to detect *M. bovis* did not yield amplifications. PCR used to detect *M. tuberculosis* yielded a specific amplicon of 389 bp, indicating the presence of *M. tuberculosis* in the lung tissue.

Nucleotide sequencing of the obtained 389-bp amplicon and subsequent phylogenetic analysis using MEGA6 software (*8*) showed 100% nt sequence identity with *M. tuberculosis* isolates deposited in GenBank (Figure, panel B), confirming the pathogen as *M. tuberculosis*. The distance map indicated that the isolate was of Indian origin. Lung samples processed and subjected for histopathologic examination in accordance with standard protocols (*9*) showed multiple granulomas, each with central caseum, surrounded by inflammatory cells and a fibrous capsule.

The gross pathology, histopathology, PCR detection, and phylogenetic analysis confirmed the infection as TB caused by the human pathogen *M. tuberculosis*. However, the uniqueness of this investigation is that this elephant had no known history of contact with humans, making the source of infection difficult to determine. Because ecologic, environmental, and demographic factors influence the emergence of disease (*10*), the infection in this elephant could be attributable to one of the following reasons. Although RGNP is an uninhabited forest, eco-tourist activities give tourists limited access to wildlife areas. Furthermore, a highway connecting 2 states, Karnataka and Kerala, passes through RGNP, enabling transit of large numbers of human through the forest. A possibility also exists that wild elephants could accidentally enter villages adjoining the forest areas in search of feed and water.

Although remote possibilities, these events can create opportunities for susceptible wildlife populations to be exposed to human pathogens. If the elephant was not infected by accidental human contacts, then it must have acquired the disease in the wild, which leads to a larger question: can wildlife species maintain and spread *M. tuberculosis* to other susceptible species in the wild?. Comprehensive studies are needed to assess the status of TB among wild animals and to examine whether wildlife can be a potential reservoir of the disease. Irrespective of source of the infection, our study indicates that elephants living in the wild can harbor *M. tuberculosis*, which can become clinical and fatal.
